# Latent variable modeling paradigms for genotype-trait association studies

**DOI:** 10.1002/bimj.201000218

**Published:** 2011-09-02

**Authors:** Yan Liu, Andrea S Foulkes

**Affiliations:** Division of Biostatistics, 404 Arnold House715 North Pleasant Street, Amherst, MA 01003, USA

**Keywords:** Mixed effects models, Single-nucleotide polymorphisms (SNPs), Structural equation model (SEM)

## Abstract

Characterizing associations among multiple single-nucleotide polymorphisms (SNPs) within and across genes, and measures of disease progression or disease status will potentially offer new insight into disease etiology and disease progression. However, this presents a significant analytic challenge due to the existence of multiple potentially informative genetic loci, as well as environmental and demographic factors, and the generally uncharacterized and complex relationships among them. Latent variable modeling approaches offer a natural framework for analysis of data arising from these population-based genetic association investigations of complex diseases as they are well-suited to uncover simultaneous effects of multiple markers. In this manuscript we describe application and performance of two such latent variable methods, namely structural equation models (SEMs) and mixed effects models (MEMs), and highlight their theoretical overlap. The relative advantages of each paradigm are investigated through simulation studies and, finally, an application to data arising from a study of anti-retroviral-associated dyslipidemia in HIV-infected individuals is provided for illustration.

## 1 Introduction

The increased availability of data on single-nucleotide polymorphisms (SNPs) has led to heighten interest in understanding how this genetic information correlates with measures of disease progression. One analytic challenge plaguing these genotype-trait association studies is the potential for multiple SNPs to be implicated in complex diseases. In this manuscript, we describe applications and performance of two latent variable paradigms, namely structural equation models (SEMs) and mixed effects models (MEMs), for addressing this challenge.

SEMs constitute a broad range of multivariate regression models that allow complex dependencies among multiple predictors and outcome variables and are widely used in economics, sociology and psychology (Pugesek et al., 2003; Rabe-Hesketh et al., 2004; Skrondal and Rabe-Hesketh, 2004). Several recent manuscripts extend the conventional measurement component of an SEM, conditional on latent variables, to the generalized linear model setting, rendering these models naturally conducive to continuous as well as categorical outcomes (Muthén, 1984; Muthén and Muthén, 2007; Skrondal and Rabe-Hesketh, 2005, 2004; Lee and Shi, 2001; Reboussin and Liang, 1998). Recent applications of SEMs to genetic data include those that aim to reconstruct the linkage disequilibrium structure among genes (Lee et al., 2007) as well as one study to characterize associations between multiple SNPs, smoking, gender and rheumatoid arthritis (Nock et al., 2007). MEMs, widely used to address correlations in repeated-measures and multi-level data (Laird and Ware, 1982), are an alternative latent variable modeling strategy that has been described for characterizing association between multiple SNPs, within and across genes, and a measured trait (Foulkes et al., 2005; Goeman et al., 2004; Foulkes and De Gruttola, 2002).

A growing body of literature exists on the methods for analyzing data arising from candidate gene association studies, including approaches targeted specifically at characterizing combinations of SNPs and their association with a measure of disease status or disease progression. Among these are most notably machine learning applications, including classification and regression trees (CART) (Zhang and Singer, 1999; Breiman et al., 1993), random forests (Bureau et al., 2005; Segal et al., 2004; Breiman, 2001), logic regression (Schwender and Ickstadt, 2008; Kooperberg and Ruczinski, 2005; Ruczinski et al., 2004, 2003; Kooperberg et al., 2001), lasso (Kooperberg et al., 2010; Wu et al., 2009; Tibshirani, 1996), elastic net (Kooperberg et al., 2010; Zou and Hastie, 2005) and Bayesian network (BN) analysis (Rodin and Boerwinkle, 2005; Pearl, 1988). The gains attributable to first-stage creation of meta-variables within these frameworks are also described, for example in Foulkes et al. (2004); Bastone et al. (2004) and Malovini et al. (2009). The former involves a first-stage, unsupervised clustering of individuals based solely on genotype data, followed by application of CART to characterize association, while the later involves a first-stage application of CART to identify clusters, followed by application of BN analysis to characterize association. The latent class approaches described herein similarly involve defining group indicators based on a collection of SNPs and, in turn, relating these to a measured trait for characterizing association; however, both the SEM and MEM approaches detailed below are distinct in that they involve fully parametric modeling of association and corresponding parameter estimation and testing. The present manuscript focuses on the overlap of these two specific latent class paradigms while additional details on several of the alternative approaches listed above, including discussion of their relative merits, can be found in Hastie et al. (2001); Gentleman et al. (2005); Schwender et al. (2008) and Foulkes (2009).

We begin by formalizing the SEM approach for genetic association studies and extend the research of Nock et al. (2007), to characterize broadly the performance under a range of underlying models of association (Section 2.1). Second, we present an extension of the MEM approach of Foulkes et al. (2005), for this setting that offers additional flexibility in defining the model of association through inclusion of cross-classified clusters (Section 2.2). We then highlight the theoretical overlap between SEMs and MEMs (Section 2.3) and explore, through simulation studies, the relative advantages of each approach (Section 3.1). Specifically, we focus on the flexibility and performance under model misspecification. Finally, we apply both approaches, as we as an alternative two-stage BN analysis, to data arising from a study of anti-retroviral therapy (ART)-associated dyslipidemia in HIV (Section 3.2).

## 2 Methods

### 2.1 Structural equation model for genetic association studies

We begin by describing how the SEM framework can be applied for analysis of data derived from genetic association studies, where the goal is to characterize associations between genotypes, within and across multiple genetic loci, and a single measure of disease progression or disease status. An extensive literature exists on SEMs, and correspondingly a variety of approaches to specifying the model have been described (Jöreskog, 1975; Bentler and Weeks, 1980; Muthén, 1984, 2002; Sánchez et al., 2005; Skrondal and Rabe-Hesketh, 2005; Muthén and Muthén, 2007). Here, we use the formulation given by Sánchez et al. (2005) and apply the measurement model described by Muthén (1984); Skrondal and Rabe-Hesketh (2005); and Muthén and Muthén (2007).

Let *y*_*i*_ denote the trait under study, where 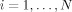 represents individuals. Further suppose 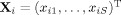 represents genotypes across *S* bi-allelic SNPs for individual *i*. Since each SNP is bi-allelic, we have 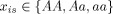, where *A* and *a* represent the two possible nucleotides at site *s* and 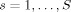. For simplicity of presentation, we drop the notational dependency of *A* and *a* on *s*. Finally, let 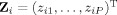 represent *P* measured covariates for individual *i*.

Similar to the approach described by Nock et al. (2007), we assume that each candidate gene has a corresponding latent variable, representing an unobservable effect of the corresponding gene on the trait. These latent variables are given for individual *i* by the vector 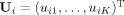 where 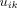 corresponds to gene *k*, 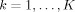. The measurement component of an SEM relates the observed data components, **X**_*i*_ and *y*_*i*_, to the latent variables, **U**_*i*_, while the structural component defines the relationship among the latent variables. These are formulated as follows:


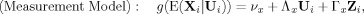
(1)


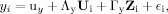
(2)

where 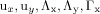 and Γ_*y*_ are unknown parameters, and 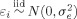. Here 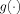 is used to represent an appropriately defined link function, such as the probit or logit link for categorical and binary outcomes, respectively


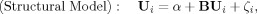
(3)

where α, **B** are unknown parameters, the diagonal elements of **B** are identically equal to 0 and (*I*−**B**) is invertible (Sánchez et al., 2005). Here we assume 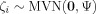 and 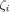 is independent of ε_*i*_. In the genetic association study setting, interest is in characterizing the association between the latent variables, **U**_*i*_, and the measured trait, given by *y*_*i*_. Formally, a test of association is given by a Wald test of the null hypothesis, 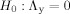.

Notably, in the genetic association setting, where **X**_*i*_ represents SNPs as described above, many of the covariates represented in **Z**_*i*_ will influence the trait *y*_*i*_ but will not be directly predictive of **X**_*i*_ as described in Eq. (1). Covariates that are potentially relevant in this component of the measure model include surrogates for population substructure, such as race and ethnicity, as well as measures of exposure to environmental toxins, such as smoking status, that may result in oncogenic mutations within specific organ tissue. In this sense, **Z**_*i*_ can be thought of as a partitioned matrix, given by 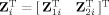, where only the covariates represented in 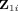 are potentially predictive of **X**_*i*_ while the covariates given in both 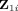 and 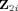 are potentially predictive of *y*_*i*_. In turn, the element of Γ_*x*_ corresponding to 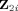 is identically equal to 0.

**Figure 1:**
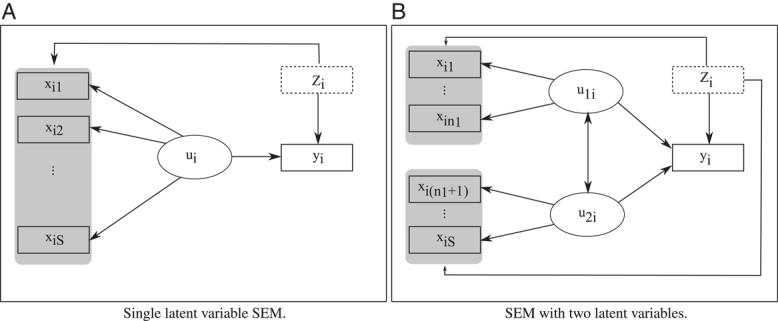
Sample SEM path diagrams for genetic association studies. (A) Single latent variable SEM and (B) SEM with two latent variables.

Visual path diagram representations of this model with one and two latent variables are given in Fig. 1A and B, respectively. Here, observed variables are represented by rectangles while latent variables are given by ovals. Dashed lines represent fixed, independent variables, whereas solid lines indicate dependent variables with corresponding distributional assumptions. Single-direction arrows represent causal relationships among variables while double-headed arrows represent non-zero correlations.

### 2.2 Mixed effect model for genetic association studies

Distinct from the SEM setting, application of an MEM to SNP data is a staged approach that involves first assigning individuals to groups based on observed genotypes across multiple SNPs. These genotype group assignments are then treated as cluster indicators in the usual mixed modeling framework. While several approaches to the first-stage dimension reduction are tenable, such as hierarchical or K-means clustering (Hartigan, 1975), here we apply the simple deterministic approach of assigning individuals with identical multi-locus genotypes to the same genotype group, as described by Foulkes et al. (2004).

Again, we begin by letting 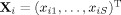 represent the multilocus genotype for individual *i* across *S* bi-allelic SNPs. Now suppose 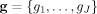 represents the *J* groups resulting from assigning individuals with identical genotypes to the same group, that is 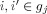 implies 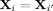. The MEM as described previously for this setting (Foulkes et al., 2005) can be formulated as follows:


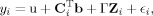
(4)

where ν and Γ are again unknown parameters, 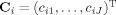, 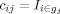 is an indicator for individual *i* belonging to genotype group *g*_*j*_, 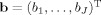 is a vector of corresponding random effects of genotype groups on the trait under study, 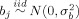 for 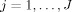, 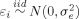 and *b*_*j*_ and ε_*i*_ are independent. A likelihood ratio test of the null hypothesis *H*_0_: σ_*b*_=0 is applied to assess the presence of a genotype-trait association.

More generally, a grouping variable can be defined for each of multiple genes. To see this, suppose now that **X**_*i*_ represents a vector of *S* SNPs across *K* genes. We assume *n*_*k*_ SNPs are measured within gene *k*, such that 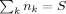. Now 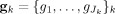 represents the groups corresponding to gene *k* where *J*_*k*_ is the number of such groups. Notably, in the setting of three-level SNPs, we have 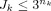, while for binary SNPs, 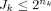. The MEM for such cross-classified data is then given by


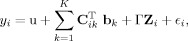
(5)

where 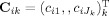 is a vector of group membership indicators, 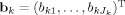 is defined as the genotype group random effects on *y*_*i*_ for gene *k*, 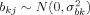 and 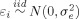. In this setting, a likelihood ratio test can again be applied to test the null hypothesis 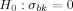 for each gene *k*.

**Figure 2:**
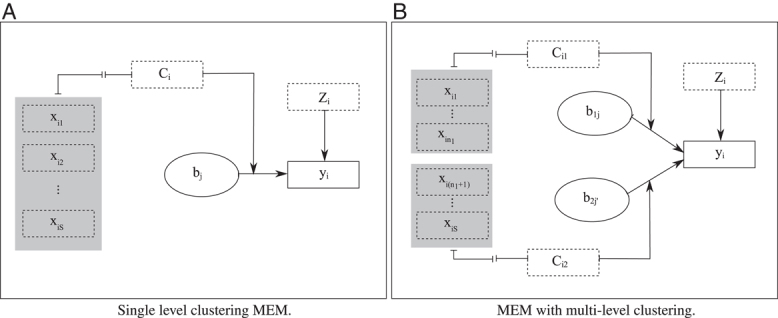
Sample MEM path diagrams for genetic association studies. (A) Single level clustering MEM and (B) MEM with multi-level clustering.

Visual representations of the MEMs for single- and multi-level clustering are given in Fig. 2A and B, using the same notation as described above for Fig. 1. Here, the broken lines indicate a deterministic relationship between SNPs – represented by 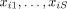 – and cluster assignments – represented by **C**_*i*_. A few notable distinctions can be discerned by comparing Figs. 1 and 2. Most notably, in the SEM framework, we see that the SNP variables are treated as random, and modeled as a function of the latent variables, *u*_*i*_. In the MEM setting, on the other hand, these are treated as fixed and inform cluster assignments deterministically. Additionally, in the SEM setting, the latent variables – given by 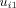 and 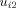 – are person-specific and potentially correlated, while in the MEM framework the latent effects – given by 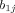 and 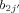 – correspond to genotype groups and are independent. Further discussion of theoretical overlap between the two modeling approaches is given in Section 2.3.

### 2.3 A comparison of the SEM and MEM approaches

Both the SEM and MEM approaches, as formulated in Sections 2.1 and 2.2, involve modeling underlying latent variables that represent unobservable effects of genes on the trait under study. Indeed, it is straightforward to show that the SEM can be reduced to an MEM for this setting. To begin, consider the simple case of a single gene, and thus a single latent variable. We first omit the regression of **X**_*i*_ on the latent variable – Eq. (1) of the SEM – as the MEM treats the **X**_*i*_ as fixed. For the single gene setting, the models of Eqs. (2) and (3) reduce to


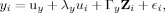
(6)


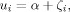
(7)

where 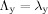 is now a scalar and, because there is only one assumed latent variable, **B** is identically equal to 0 and 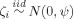. In order for this SEM to reduce to the MEM, we let λ_*y*_ equal the vector 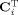 and replace the individual level latent variable *u*_*i*_ with the vector of random cluster effects 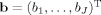. Importantly, this is equivalent to making the assumption that the latent effects on the trait are the same for those individuals with the same observed genetic profile. Finally, we set α=0 and, together, these restrictions yield Eq. (4).

In the case of *K*=2 genes, we note that Eqs. (2) and (3) can be written as:


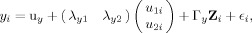
(8)


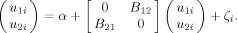
(9)

Now we replace 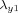 and 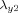 with the vectors 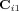 and 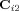 and replace 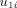 and 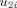 with 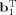 and 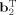, respectively. Notably, as the lengths of 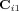 and 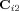 (as well as **b**_1_ and **b**_2_), given by *J*_1_ and *J*_2_, are not necessarily equal, these vectors need to be concatenated with vectors of 0 of appropriate length. That is, Eq. (8) is replaced with:


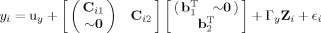


where, without loss of generality, we assume *J*_1_<*J*_2_ and 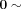 is defined as a vector of length (*J*_2_−*J*_1_) with all 0 elements. In order for this SEM to simplify to the MEM with two levels of clustering, we additionally need to assume α=0 and 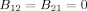. In other words, we must assume that the latent variables are uncorrelated and centered at 0.

In summary, and more generally for *K*>2 genes, we make the following three assumptions for the SEM to reduce to the MEM: (i) SNPs, represented by **X**_*i*_, are fixed, so that the model of Eq. (1) is omitted; (ii) Λ_*y*_**U**_*i*_ is given by 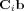, that is individual level latent variables are the same for individuals within the same defined genotype group and (iii) α=**B**=0, that is latent variables across genes are mutually independent and centered at 0. In the MEM setting, the cluster random effects are assumed independent, although a correlation structure between **b**_*j*_ and 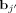 could be imposed.

## 3 Applications

In the following sections we report the results of a simulation study and an application to a study of anti-retroviral-associated dyslipidemia in HIV. Restricted maximum likelihood is used to derive point estimates of parameters in the MEMs. A likelihood ratio test of 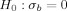, comparing the full (mixed effects) and reduced (fixed effects only) model, is used to investigate the association between genotype groups and a measured trait. As this involves testing a parameter at a boundary, a mixture of a χ^2^ with 1 and 0 degrees of freedom is assumed for the resultant test statistic. All MEMs are fitted with the lme() function within the nlme package in R, Version 2.9.1. In the context of fitting SEMs, weighted least squares is applied to derive parameter estimates and a Wald test of the null hypothesis 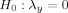, is reported. SEMs are fitted using Mplus Version 5.21.

### 3.1 Simulation studies

In this section we explore, through simulation studies, the relative practical performance of SEMs and MEMs under a range of underlying models of association. In each simulation, we generate 500 sets of data, each of size *n*=1000, for each combination of true parameter values. SEM data are simulated using the MONTECARLO Command in Mplus Version 5.21 (Muthén, 1984; Muthén and Muthén, 2007).

We begin by simulating data under an SEM with a single latent variable, according to Eqs. (1) and (6)–(7), where for simplicity of presentation, we let Γ_*x*_=0. In scalar notation, this model can be rewritten as:


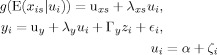


where we assume 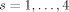, *x*_is_ is a binary SNP indicator, 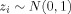, 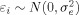, 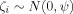 and 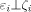. For identifiability, α is set to 0 and 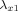 is restricted to 1. Furthermore, we define a threshold model in Mplus such that 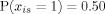. It is straightforward to show that the covariance between any two SNPs is then given by ψ. Finally, we set 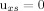, 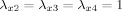 and 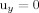, while the values of the remaining parameters, λ_*y*_, 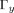, 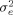, and ψ, vary as described in Table 1.

**Table 1:** Simulation results under SEM with one latent variable.

Data	True value	SEMs				MEMs			
			
		Bias	CR	CI	Power	Bias	CR	CI	Power
1	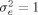	0.00	0.95	0.23	–	0.13	0.24	0.20	–
	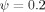	0.01	0.96	0.21	–	–	–	–	–
	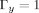	0.00	0.95	0.14	–	0.00	0.96	0.13	–
	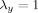	−0.02	0.94	0.73	1.00	–	–	–	1.00
2	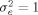	0.00	0.95	0.23	–	0.18	0.06	0.21	–
	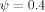	0.01	0.96	0.20	–	–	–	–	–
	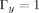	0.00	0.95	0.15	–	0.00	0.96	0.14	–
	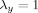	−0.01	0.95	0.37	1.00	–	–	–	1.00
3	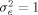	0.00	0.94	0.22	–	0.19	0.03	0.21	–
	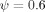	0.01	0.96	0.17	–	–	–	–	–
	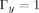	0.00	0.95	0.16	–	0.00	0.95	0.14	–
	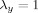	0.00	0.97	0.25	1.00	–	–	–	1.00
4	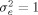	0.00	0.94	0.19	–	0.04	0.85	0.18	–
	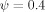	0.01	0.95	0.22	–	–	–	–	–
	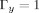	0.00	0.96	0.13	–	0.00	0.96	0.13	–
	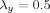	−0.01	0.96	0.30	1.00	–	–	–	1.00
5	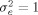	0.01	0.95	0.39	–	0.69	0.00	0.30	–
	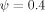	0.00	0.96	0.18	–	–	–	–	–
	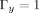	0.00	0.95	0.20	–	0.00	0.95	0.16	–
	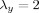	−0.02	0.95	0.56	1.00	–	–	–	1.00

(a) Median estimates are reported from 500 sets of data. (b) Absolute difference between Est. and true value. (c) Coverage rate, the percentage of confidence intervals (CIs) that cover true value among 500 CIs. For the CR of variance, the CIs that contain negative values are excluded for consideration. (d) Median length among the 500 length of CIs. (e) Wald test statistics of 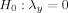 is used to test the association between latent variable and measured trait. (f) Likelihood ratio test (LRT) of 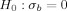 is applied to investigate the association between genotype groups and measured trait. Here the approximate distribution of LRT under null hypothesis is 50:50 mixture of 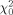 and 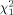.

The results of fitting both SEMs and MEMs to these data are provided in Table 1. In general, both approaches have high power for detecting association, but the SEM approach performs better in terms of bias and coverage for the variance component, 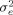. As ψ and λ_*y*_ increase while 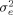 remains fixed, the absolute bias associated with 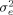 for the MEM increases and the corresponding coverage rate (CR) is low. Notably, the type-1 error rate (under the model in which λ_*y*_=0) is 0.03 for both the SEM and MEM approaches.

Second, we simulate data according to an SEM with two uncorrelated latent variables. That is, we let the data arise from Eqs. (1) and (8)–(9) with *B*=0. In this case, our model can be written as:


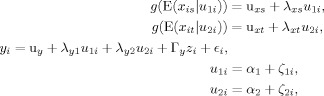


where 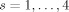 and 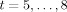, that is we have 8 SNPs with 4 in each of two genes. We further assume 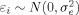, 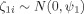, 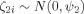, all mutually independent and 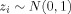. For identification, α_1_ and α_2_ are set to 0 and we restrict 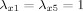. For the simulation, we set 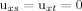, 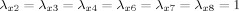, 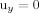, and define a threshold in Mplus such that 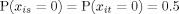. Finally, the values of 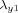, 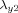, γ_*y*_, 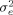, ψ_1_, and ψ_2_ are assumed to vary as described in Table 2.

**Table 2:** Simulation results under SEM with two uncorrelated latent variables.

Data	True value	SEMs				MEMs			
			
		Bias	CR	CI	Power	Bias	CR	CI	Power
1	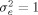	−0.01	0.94	0.30	–	0.25	0.00	0.22	–
	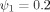	0.00	0.95	0.21	–	–	–	–	–
	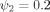	0.00	0.94	0.21	–	–	–	–	–
	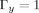	0.00	0.94	0.15	–	0.00	0.95	0.14	–
	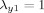	0.00	0.96	0.81	1.00	–	–	–	1.00
	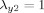	0.00	0.96	0.82	1.00	–	–	–	1.00
2	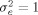	0.00	0.94	0.28	–	0.34	0.00	0.24	–
	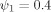	0.00	0.96	0.20	–	–	–	–	–
	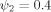	0.00	0.94	0.20	–	–	–	–	–
	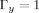	0.00	0.95	0.17	–	0.00	0.93	0.15	–
	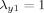	0.00	0.97	0.41	1.00	–	–	–	1.00
	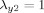	0.00	0.94	0.41	1.00	–	–	–	1.00
3	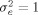	0.00	0.93	0.27	–	0.37	0.00	0.24	–
	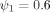	0.00	0.94	0.18	–	–	–	–	–
	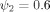	0.00	0.94	0.17	–	–	–	–	–
	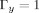	0.00	0.95	0.18	–	0.00	0.94	0.15	–
	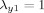	0.00	0.97	0.27	1.00	–	–	–	1.00
	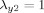	0.00	0.93	0.27	1.00	–	–	–	1.00
4	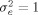	0.00	0.94	0.61	–	1.37	0.00	0.42	–
	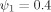	0.00	0.95	0.19	–	–	–	–	–
	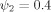	0.00	0.93	0.19	–	–	–	–	–
	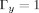	0.00	0.96	0.25	–	0.00	0.94	0.19	–
	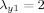	−0.01	0.96	0.67	1.00	–	–	–	1.00
	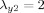	−0.01	0.94	0.66	1.00	–	–	–	1.00
5	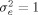	0.00	0.95	0.41	–	0.72	0.00	0.31	–
	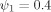	0.00	0.95	0.22	–	–	–	–	–
	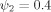	0.00	0.94	0.18	–	–	–	–	–
	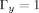	0.00	0.95	0.20	–	0.00	0.95	0.16	–
	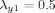	0.00	0.97	0.41	1.00	–	–	–	1.00
	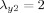	−0.01	0.93	0.57	1.00	–	–	–	1.00

The results of fitting the SEM and MEM to these data are given in Table 2. The results are similar to those we saw with a single latent variable, with more extreme biases associated with 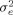 using the MEM approach. The type-1 error rates in the SEM setting are 0.05 and 0.06 for 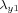 and 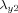, respectively, while the type-1 error rates in the MEM setting are 0.04 and 0.05 for 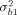 and 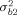, respectively.

Finally, we simulate data according to an SEM with two correlated latent variables, where the model is the same as described above for two uncorrelated latent variables, with the exception that it is assumed 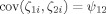. Data are simulated under two models specified by ψ_12_=0.1 and ψ_12_=0.2, where in both cases ψ_1_=ψ_2_=0.4. These models correspond to correlations between latent variables of 0.25 and 0.5, respectively. The results are reported in Table 3. Again power is high under both the SEM and MEM, and similar bias is observed under the MEM for 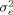. The corresponding type-1 error rates are 0.05 and 0.06 for both SEM parameters, 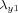 and 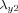, and 0.04 and 0.05 for the two MEM variance parameters.

**Table 3:** Simulation results under SEM with two correlated latent variables.

Data	True value	SEMs				MEMs			
			
		Bias	CR	CI	Power	Bias	CR	CI	Power
1	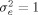	0.00	0.93	0.28	–	0.37	0.00	0.24	–
	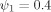	0.00	0.95	0.20	–	–	–	–	–
	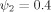	0.00	0.94	0.20	–	–	–	–	–
	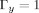	0.00	0.95	0.18	–	0.00	0.93	0.15	–
	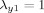	0.00	0.94	0.42	1.00	–	–	–	1.00
	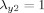	−0.01	0.93	0.42	1.00	–	–	–	1.00
	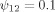	0.00	0.96	0.09	–	–	–	–	–
2	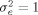	0.00	0.93	0.28	–	0.40	0.00	0.25	–
	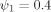	0.00	0.94	0.20	–	–	–	–	–
	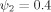	0.00	0.94	0.20	–	–	–	–	–
	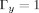	0.00	0.95	0.18	–	0.00	0.94	0.15	–
	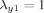	0.00	0.95	0.49	1.00	–	–	–	1.00
	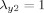	0.00	0.95	0.49	1.00	–	–	–	1.00
	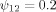	0.00	0.95	0.10	–	–	–	–	–

Next we simulate data under an MEM with a single clustering variable, as described by Eq. (4). We again assume *S*=4 SNPs, each coded as binary indicators with 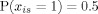 and minimal correlation induced by the assumption 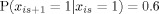 and 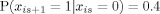 for *s*=1,2,3. A single continuous covariate 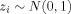 is generated and we set ν=0. The remaining parameters, 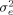, 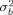 and γ are varied across the simulations as given in Table 4. The results of fitting SEMs and MEMs to these data are reported in Table 4. In this setting, a test of λ_*y*_=0 has reduced power for detecting association. The type-1 error rates are 0.05 and 0.03 for the SEM and MEM approaches, respectively.

**Table 4:** Simulation results under MEM with single-level clustering.

Data	True value	SEMs				MEMs			
			
		Bias	CR	CI	Power	Bias	CR	CI	Power
1	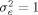	0.12	0.42	0.21	–	0.00	0.96	0.18	–
	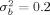	–	–	–	0.65	−0.01	0.94	0.33	1.00
	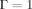	0.00	0.93	0.13	–	0.00	0.94	0.13	–
2	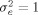	0.22	0.23	0.25	–	0.00	0.94	0.18	–
	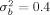	–	–	–	0.76	0.00	0.95	0.65	1.00
	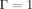	0.00	0.94	0.15	–	0.00	0.95	0.13	–
3	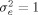	0.33	0.17	0.28	–	0.00	0.94	0.18	–
	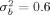	–	–	–	0.77	−0.02	0.95	0.94	1.00
	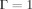	0.00	0.95	0.15	–	0.00	0.94	0.12	–

Finally, we generate data according to a two-level clustering MEM, as described by Eq. (5). Here we again assume that we observe 4 SNPs in each of 2 genes. The corresponding results of fitting SEMs and MEMs to these data are given in Table 5. Again the power for the SEM approach to detect association based on a test of λ_*y*_ is relatively small in all cases while the power for the MEM approach is reasonable (>90%) for 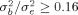. The estimated type-1 error rates are 0.03 and 0.05 for the SEM parameters and 0.01 for both of the MEM parameters.

**Table 5:** Simulation results under MEM with two-level clustering.

Data	True value	SEMs				MEMs			
			
		Bias	CR	CI	Power	Bias	CR	CI	Power
1	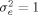	0.31	0.03	0.23	–	0.00	0.95	0.18	–
	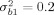	–	–	–	0.65	−0.01	0.98	0.45	0.97
	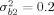	–	–	–	0.68	−0.01	0.96	0.44	0.97
	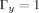	0.00	0.94	0.15	–	−0.01	0.94	0.25	–
2	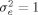	0.59	0.00	0.29	–	0.00	0.94	0.18	–
	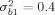	–	–	–	0.78	−0.01	0.95	0.79	0.99
	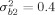	–	–	–	0.79	−0.02	0.96	0.77	1.00
	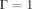	0.00	0.94	0.16	–	0.00	0.94	0.25	–
3	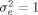	0.95	0.00	0.36	–	0.00	0.95	0.18	–
	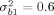	–	–	–	0.77	−0.03	0.95	1.10	1.00
	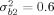	–	–	–	0.77	0.00	0.95	1.11	0.99
	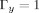	0.00	0.95	0.18	–	0.00	0.94	0.25	–
4	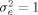	0.47	0.00	0.27	–	0.00	0.97	0.18	–
	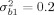	–	–	–	0.73	−0.01	0.97	0.48	0.97
	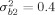	–	–	–	0.74	−0.02	0.96	0.73	1.00
	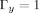	0.00	0.96	0.15	–	0.00	0.96	0.25	–

### 3.2 Genetics of therapy-associated lipid abnormalities in HIV

In this section we apply the SEM and MEM frameworks to data arising from the New Works Concept Sheet (NWCS) 224 study, an investigation of genetic factors that contribute to anti-retroviral-associated dyslipidemia in HIV-1-infected individuals. This cross-sectional study is comprised of *n*=626 HIV-infected participants enrolled in 5 AIDS Clinical Trials Group (ACTG) trials who agreed to genetic testing. A complete discussion of the study design and patient demographics is given in Foulkes et al. (2006). Here we focus on 7 SNPs – rs1045642, rs2032582, rs2235035, rs11772987, rs10256836, rs9282564 and rs2157926 – within the ATP-binding cassette, sub-family B (MDR/TAP), member 1 (ABCB1) gene, a gene involved in transporting substrates, including Protease Inhibitors (PIs) across the cell membrane, and an additional 3 SNPs – rs2854117, rs4520 and rs2070669 – in apolipoprotein C-III (APOC3), a gene involved in inhibiting hepatic uptake of triglyceride-rich particles. All SNPs are treated as binary indicators for the presence of at least one variant allele. Interest is in characterizing association between these SNPs and high-density lipoprotein cholesterol (HDL-C). White/non-Hispanic, Hispanic and Black/non-Hispanic subjects (*n*=532) with complete data, including known drug exposure histories, are included in analysis.

The results of fitting unadjusted models are reported in Table 6. Here we consider three models: two single-gene models (that include either ABCB1 or APOC3) and one two-gene model (that includes both ABCB1 and APOC3). The SEM and MEM results are consistent with one another, suggesting that ABCB1 is associated with HDL-C, as measured by 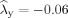 (*p*=0.002 and *p*=0.003) in the SEM and 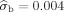 (*p*=0.02) in the MEM for both the single-gene and two-gene models. These effects are attenuated (and no longer statistically significant) in adjusted models and may represent spurious associations resulting from population-admixture, i.e. confounding by race/ethnicity and study site. Adjusted models also included PI exposure as a three-level factor – no current PI exposure; currently exposed to a non-RTV-containing PI regimen; and currently exposed to an RTV-containing PI regimen – gender, race/ethnicity and study.

**Table 6:** Application to study of therapy-associated lipid abnormalities in HIV.

	ABCB1 – model		APOC3 – model		(ABCB1, APOC3) – model	
			
	SEM	MEM	SEM	MEM	SEM	MEM
	Est (*p*-value)	Est (*p*-value)	Est (*p*-value)	Est (*p*-value)	Est (*p*-value)	Est (*p*-value)
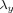	−0.06 (0.002)	–	0.02 (0.53)	–	−0.06 (0.003); 0.01 (0.84)	–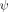	0.78	–	0.18	–	0.78, 0.28	–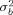	–	0.004 (0.02)	–	0.00 (0.50)	–	0.004 (0.02); 0.00 (0.50)
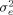	0.097	0.096	0.099	0.099	0.097	0.096

To further explore the results of this model fitting procedure, we consider 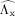 from the SEM and the empirical Bayes estimates of the random effects from the MEM. For illustration, we focus on the unadjusted model with the single-gene ABCB1. Based on the SEM, the relationship between the SNPs and the latent gene variable, *u*_*i*_, is estimated by 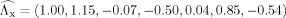 corresponding to rs1045642, rs2032582, rs2235035, rs11772987, rs10256836, rs9282564 and rs2157926, respectively. Associated *p*-values based on a Wald test are given by 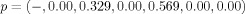 where *p*-values of “0” are less than 1×10^−8^. Recall the first element of Λ is fixed at 1 for identifiability. These results suggest variant alleles at rs2032582 (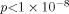) and rs9282564 (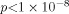) are significantly positively associated with *u*_*i*_, while variant alleles at rs11772987 (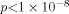) and rs2157926 (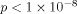) are significantly negatively associated with *u*_*i*_. Further, 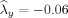, suggesting an inverse relationship between *u*_*i*_ and HDL-C.

**Figure 3:**
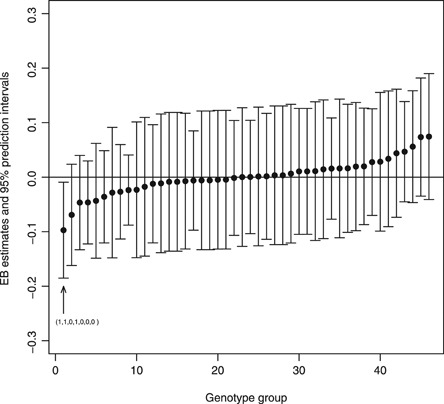
Empirical Bayes (EB) estimates of latent genotype group effects.

In total there are 46 observed genotype groups and the corresponding empirical Bayes estimates based on the MEM range from −0.097 to 0.074, as illustrated in Figure 3. All corresponding 95% prediction intervals for the genotype groups cover zero, with the exception of the group with at least one variant allele at each of the three SNPs, rs1045642, rs2032582 and rs11772987 and homozygous for the common allele at all other SNPs. Membership to this group is inversely associated with HDL-C with a corresponding empirical Bayes estimate of −0.097 and a 95% prediction interval of (−0.185,−0.009).

### 3.3 An alternative Bayesian network analysis framework

BN analysis is an alternative analysis framework that similarly aims to identify and characterize association among combinations of potential predictor variables and an observed trait. In this section, we briefly describe the application of one such approach, proposed by Malovini et al. (2009). This strategy is comprised of two stages: First, meta-variables are created based on fitting a classification or regression tree to the trait under study where SNPs and other potentially relevant variables are treated as potential predictor variables; and second, a BN is learned based on these meta-variables and the trait under study. Analysis is based on application of the rpart() and network() functions within the R rpart and deal packages, respectively.

**Figure 4:**
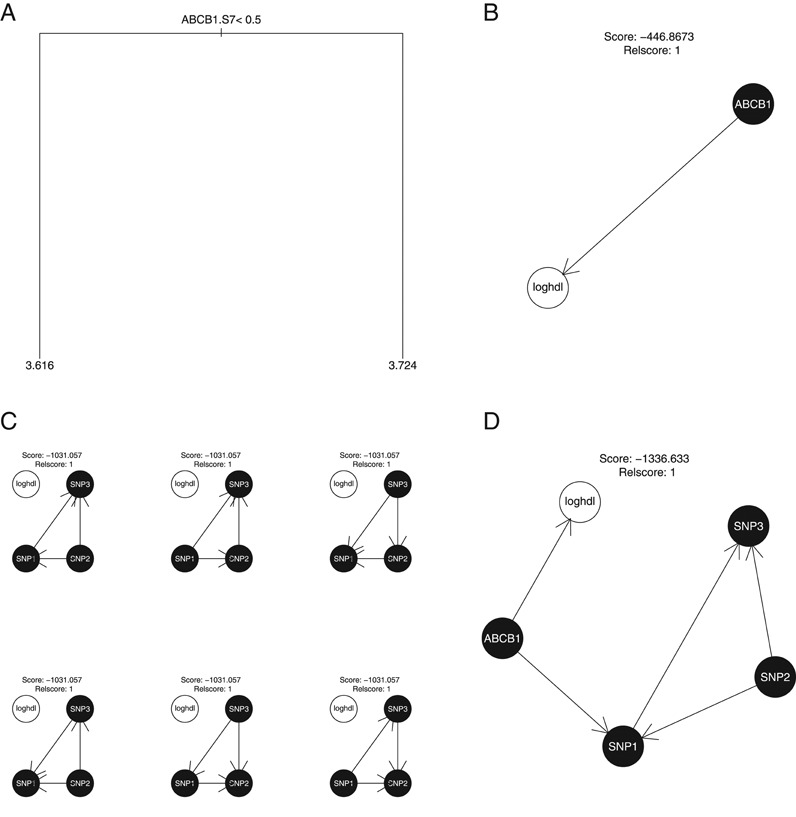
Bayesian networks of ABCB1, APOC3 and HDL-C. (A) Fitted regression tree using all SNPs within ABCB1 (coded as binary indicators for the presence of at least one variant allele) as potential predictor variables and log-transformed HDL-C as the outcome. (B) DAG based on ABCB1 metavariable and log-transformed HDLC. (C) DAGs with highest scores based on individual SNPs within APOC3 and log-transformed HDL-C. (D) DAG based on ABCB1 metavariable, individuals SNPs within APOC3 and log-transformed HDL-C.

The results of applying the approach of Malovini et al. (2009), to the SNPs within ABCB1 and APOC3 separately, and in combination, are provided in Fig. 4. To begin, we fitted a regression tree to the log transformed quantitative trait, HDL-C separately for each gene. For ABCB1, a single split is observed, as illustrated in Fig. 4A, where ABCB1.S7 corresponds to rs2157926, and a cost complexity parameter (cp) of 0.01 is applied for first-stage pruning. This constitutes the metavariable used for the second-stage BN analysis of ABCB1. For APOC3, no splits result in an increase of more than cp=0.01 in the overall R-squared value, and thus individual SNP variables for this gene are used in the second-stage BN analysis. The resulting directed acyclic graphs (DAGs) illustrated in Fig. 4B–D are consistent with the results presented in Table 6 based on the SEM and MEM analyses. Again, an association between ABCB1 and HDL-C is observed (Fig. 4B), while associations between SNPs within APOC3 and HDL-C are not detectable (Fig. 4C). The DAG based on both genes (Fig. 4D) additionally identifies an association between ABCB1 and the first SNP within APOC3.

**Figure 5:**
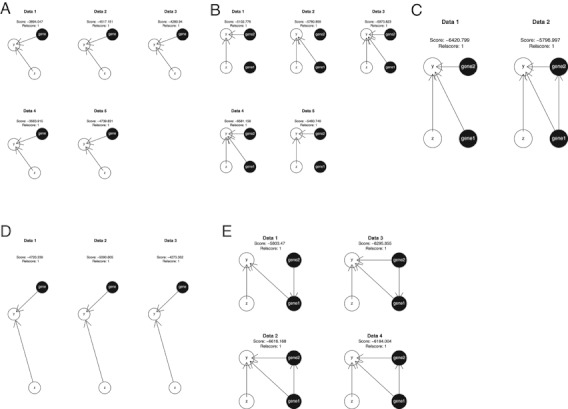
Bayesian networks of simulated data. (A) DAGs based on data simulated according to a SEM with one latent variable where gene represents a metavariable based on a fitted regression tree. The 5 DAGs correspond to each combination of parameters given in Table 1. (B) DAGs based on data simulated according to a SEM with two uncorrelated latent variables where gene1 and gene2 represent metavariables based on two separate fitted regression trees. The 5 DAGs correspond to each combination of parameters given in Table 2. (C) DAGs based on data simulated according to a SEM with two correlated latent variables where gene1 and gene2 represent metavariables based on two separate fitted regression trees. The 2 DAGs correspond to each combination of parameters given in Table 3. (D) DAGs based on data simulated according to an MEM with single-level clustering where gene represents a metavariable based on a fitted regression tree. The 3 DAGs correspond to each combination of parameters given in Table 4. (E) DAGs based on data simulated according to an MEM with two-level clustering where gene1 and gene2 represent metavariables based on two separate fitted regression trees. The 4 DAGs correspond to each combination of parameters given in Table 5.

Finally, as a case study, we applied the two-stage BN approach to a single randomly selected simulated data set from each of the scenarios (i.e. combinations of parameters) described in Tables 1–5. The results are presented in Fig. 5. In-line with the finding presented in Table 1 of consistently high power of the SEM and MEM under the SEM model with a single latent variable, Fig. 5A illustrates that all five corresponding DAGs identify association between the gene metavariable and the trait. Under the SEMs with two uncorrelated latent variables, the BN analysis consistently identifies at least one of the two gene metavariables; however, in 3 of the 5 cases only one gene is identified, as shown in Fig. 5B. The association between the two genes under the SEM with correlated latent variables is detected with higher correlation, as illustrated in Fig. 5C. Finally, the BN analysis appropriately identifies the gene metavariable for the MEM with single-level clustering, and, in 3 of the 4 cases, was able to identify both gene metavariables under the MEM with two-level clustering.

## 4 Discussion

In this manuscript we describe the application of two related latent variable modeling approaches, MEMs and SEMs, for identifying and characterizing genetic contributors to complex diseases. While these two frameworks have some important commonalities, several notable differences emerged during our investigation. These are highlighted by the assumptions listed in Section 2.3, under which the SEM reduces to the MEM as described for this setting. Importantly, in the context of an SEM, the test of association is based on a fixed effect coefficient (λ_*y*_) relating the latent gene variable to the trait. In the MEM context, on the other hand, the test of association is based on a variance parameter (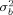) of the latent gene effects.

Interestingly, our simulation studies reveal that the performance of the two modeling approaches is comparable under the SEMs in terms of power and type-1 error rates; however, when the data arise from an MEM, power for the SEM approach is lower than the corresponding power for detecting association using the MEM approach. Also of note, when data are simulated under the SEM model, the estimates of the nuisance parameter, σ_*e*_, under the MEM exhibit substantial bias. In these cases, the estimate of σ_*b*_ is also estimated poorly (results not shown). Notably, for Tables 1 and 2, var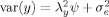 and an estimate of this variance under the MEM is 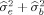. For example, for the first scenario in Table 1, we have 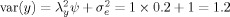 and the estimate under the MEM is 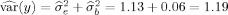 (results not shown). In general, these are not as closely aligned; however, there appears to be a tradeoff between the two parameters. In turn, estimation of γ_*y*_ depends on 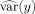. Further research on sensitivity to alternative underlying model specifications may further elucidate the relative merits of each approach. Specifically, SEMs may be more conducive to testing specific hypotheses involving multiple genes and their relationships to one another in a pathway to disease.

A notable limitation of both the MEM and SEM approaches is their potential inability to handle a large number of SNPs. In the context of the MEM, the number of genotype groups can become unwieldy as the number of SNPs increases, as described by Foulkes et al. (2008). Interestingly, inclusion of highly correlated SNPs in the MEM approach results in fewer genotype groups but does not otherwise effect model performance. Furthermore, our preliminary research suggests that iteratively sampling a subset of SNPs and fitting the MEM, and then combining results over the multiple samples (Efron, 1979, 1981; Good, 2005), performs reasonably well (results not shown) in terms of the overall power for detecting association in the setting of a large number of SNPs. The extension of the MEM approach to more than one clustering variable, as described in Section 2.2, also offers the advantage of reducing the total number of genotype groups. For example, if we observe *r* SNPs in one gene and *s* SNPs in a second gene, the original formulation of the MEM approach involves 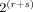 genetic groups while the proposed extension involves only 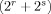 genetic groups (across two clustering variables). Additional research is needed to evaluate the performance of this extended MEM approach with multiple SNPs across a larger number of genes.

We also presented the results of applying an alternative two-stage BN analysis approach to the NWCS224 data, as well as to randomly selected simulated data sets. In the real data example, we were unable to fit a regression tree with splits beyond the root node subject to the specified pruning criterion for one of the genes under study. In this case, the analysis reduced to fitting a BN to single SNP variables rather than metavariables as described by Malovini et al. (2009). On the other hand, for the ABCB1 gene, a metavariable did emerge, albeit based on a single SNP, and an association was detected. Importantly, the structure of association that CART is designed to detect, namely conditional associations (Foulkes, 2009), may explain why only a single SNP emerged while for the SEM four SNPs within this gene were identified as statistically relevant. Although our case studies of simulated data suggest that the BN analysis generally (though not always consistently) identified relevant genes, further investigation is needed to characterize the overall performance and type-1 error rates.

Finally, further research is needed to characterize the performance of both the SEM and MEM frameworks in the context of more complex structures of association and underlying genetic models. In a recent manuscript, we describe application of a mixture modeling approach that may be more appropriate than the single normal prior assumption on the random effects under a dominant or recessive genetic model (Au et al., 2010). While the inclusion of cross-classified clusters, as proposed in Section 2.2, allows for consideration of more SNPs through reduction in the number of genotype groups, this approach assumes an additive model of association. More generally, it is of interest to characterize interactions among genes in their effect on the trait under study, an area of on-going research.

## Supplementary material

Detailed facts of importance to specialist readers are published as ”Supporting Information”. Such documents are peer-reviewed, but not copy-edited or typeset. They are made available as submitted by the authors.
